# Generalist Taxa Shape Fungal Community Structure in Cropping Ecosystems

**DOI:** 10.3389/fmicb.2021.678290

**Published:** 2021-07-09

**Authors:** Jun-Tao Wang, Ju-Pei Shen, Li-Mei Zhang, Brajesh K. Singh, Manuel Delgado-Baquerizo, Hang-Wei Hu, Li-Li Han, Wen-Xue Wei, Yun-Ting Fang, Ji-Zheng He

**Affiliations:** ^1^State Key Laboratory of Urban and Regional Ecology, Research Center for Eco-Environmental Sciences, Chinese Academy of Sciences, Beijing, China; ^2^Hawkesbury Institute for the Environment, Western Sydney University, Penrith, NSW, Australia; ^3^University of Chinese Academy of Sciences, Beijing, China; ^4^Global Centre for Land-Based Innovation, Western Sydney University, Penrith, NSW, Australia; ^5^Cooperative Institute for Research in Environmental Sciences, University of Colorado, Boulder, CO, United States; ^6^Faculty of Veterinary and Agricultural Sciences, The University of Melbourne, Parkville, VIC, Australia; ^7^Key Laboratory for Humid Subtropical Eco-Geographical Processes of the Ministry of Education, Fujian Normal University, Fuzhou, China; ^8^Key Laboratory of Agro-Ecological Processes in Subtropical Region, Institute of Subtropical Agriculture, Chinese Academy of Sciences, Changsha, China; ^9^CAS Key Laboratory of Forest Ecology and Management, Institute of Applied Ecology, Chinese Academy of Sciences, Shenyang, China

**Keywords:** coexistence pattern, niche differentiation, functional traits, soil fungi, community structure, cropland soil, ecological network

## Abstract

Fungi regulate nutrient cycling, decomposition, symbiosis, and pathogenicity in cropland soils. However, the relative importance of generalist and specialist taxa in structuring soil fungal community remains largely unresolved. We hypothesized that generalist fungi, which are adaptable to various environmental conditions, could potentially dominate the community and become the basis for fungal coexisting networks in cropping systems. In this study, we identified the generalist and habitat specialist fungi in cropland soils across a 2,200 kms environmental gradient, including three bioclimatic regions (subtropical, warm temperate, and temperate). A few fungal taxa in our database were classified as generalist taxa (~1%). These generalists accounted for >35% of the relative abundance of all fungal populations, and most of them are Ascomycota and potentially pathotrophic. Compared to the specialist taxa (5–17% of all phylotypes in three regions), generalists had a higher degree of connectivity and were often identified as hub within the network. Structural equation modeling provided further evidence that after accounting for spatial and climatic/edaphic factors, generalists had larger contributions to the fungal coexistence pattern than habitat specialists. Taken together, our study provided evidence that generalist taxa are crucial components for fungal community structure. The knowledge of generalists can provide important implication for understanding the ecological preference of fungal groups in cropland systems.

## Background

Soil microorganisms are indispensable components for sustainable agricultural production and food security for growing world populations (Bender et al., 2016). As one of the most abundant soil microbial groups, fungi possess critical functional traits that are essential for regulating plant productivity, nutrient cycling, and pathogenicity in croplands worldwide (Croll and McDonald, 2017; Wei et al., 2017). For example, saprophytic fungi regulate decomposition and mycorrhizal fungi are mutualistic symbionts with crops, while typical plant pathogens can result in substantial yield reductions (Bender et al., 2016). Different functional groups in the community generate complex coexistence patterns due to environmental preferences and potential interactions among different taxa (Wang et al., 2015; Purahong et al., 2016). However, how the coexistence pattern of multiple taxa determines soil fungal community structure in large-scale agricultural ecosystems remains largely unexplored.

Niche differentiation is an important ecological strategy that can lead to the coexistence patterns among different species (Devictor et al., 2008). Generalist fungi usually have broad habitat niches and can be more resistant to disturbances and/or changes in resource and habitat availability. Therefore, they are expected to be found across heterogeneous environments and resistant to variations in soil properties and climate. For example, a few Ascomycota that accounted merely 0.1% of the total phylotypes dominated various natural ecosystems at the global scale (Egidi et al., 2019), and these generalists had more traits relating to stress tolerance and resource uptake. By contrast, specialist fungi have a narrow ecological niche and can only use limited resource. They are usually considered to be less tolerant to environmental disturbance. Typical specialist fungi include some saprophytic wood-inhabiting fungi and mycorrhizal fungi that specifically adhered to certain plants (Nordén et al., 2013).

Different from the natural ecosystems, croplands are artificial systems that can be strongly affected by multiple human activities, e.g., synthesized/organic fertilizer inputs, pesticide/herbicide usage, tillage, irrigation, and cropping. These activities greatly changed the resources and environment of soil fungi and thereafter their communities (Zhou et al., 2016; Hu et al., 2020). Fertilization is the most recognized factor on soil fungal communities since it strongly changed soil nutrients and carbon conditions; some fungal taxa are known to be highly resistant to the disturbance caused by agricultural practices (Sun et al., 2016). They could be important in evaluating the patterns of dominant fungi on a large scale (i.e., generalist) and have significant implications for the understanding of the soil processes in croplands. However, we lack empirical evidence and information on the generalist fungal taxa found in croplands across wide environmental gradients.

Here, we aimed to (1) identify the taxa and functional traits of generalist fungi across continental scale cropland ecosystems and (2) evaluate the relative importance of generalists vs. specialists in regulating the structure of fungal communities. To achieve these aims, we collected 197 soil samples from croplands (mostly corn-wheat rotations; corn was grown at the time of sampling) across a 2,200 kms distance in eastern China and used amplicon sequencing and network analysis to characterize the soil fungal community structure. We hypothesized that generalist fungi are likely to be dominant across environmental gradients and may potentially constitute the core structure of fungal ecological network at a large spatial scale, while habitat specialist fungi can be much less dominant across large spatial scale but might be more adaptive to the specific environment. Knowledge of generalist/specialist fungi can help us to develop strategies to predict how fungal communities respond to intensifying human activities in agricultural ecosystems and provide basic information to improve fungi-mediated functions in agricultural soils, e.g., constituting the core components of root microbiome (Banerjee et al., 2019).

## Materials and Methods

### Study Sites and Soil Sampling

The study covered 2,200 kms across the latitudinal gradient in eastern China (26.39°E to 46.33°E, 111.24°N to 126.92°N). From south to north, there are subtropical, warm temperate, and temperate regions according to the Köppen-Geiger climate classification system (Peel et al., 2007). Sampling sites located in cropland areas within the three climate regions. Detailed sampling information is shown in Supplementary Figure S1. Briefly, ten locations, including Qiyang (QY), Yingtan (YT), Taoyuan (TY), Zhoukou (ZK), Fengqiu (FQ), Dezhou (DZ), Luancheng (LC), Gongzhuling, Changchun (CC), and Harbin, were selected for soil sampling during July and August in 2014. At each location, 15–30 sites with 5 to 10 kms from each other were selected and five soil cores were collected and mixed into one composite sample at each site. A total of 197 soil samples were obtained for this study. Soil samples were sieved through a 2 mm mesh after removing residuals and roots, and divided into two parts: one for soil biogeochemical analysis stored at 4°C and one for DNA extraction at −20°C.

### Measurement of Soil Biogeochemical Properties

Soil biogeochemical properties were measured as previously described before (Du et al., 2020). Briefly, total nitrogen (TN) was measured using an Elementar Vario EL III analyzer (Elementar Analysen System GmbH, Germany). Soil organic carbon (SOC) was determined by the K_2_Cr_2_O_7_ oxidation method, and dissolved organic carbon (DOC; extracted with the 0.5 M K_2_SO_4_ solution) was measured using a Shimadzu organic carbon analyzer (Shimadzu, Kyoto, Japan). Soil pH was measured by a Delta pH meter with a soil to water ratio at 1:2.5. Soil nitrate and ammonium were extracted by 1.0 M KCl and measured using a SKALAR continuous flow analyzer (Skalar Analytical BV, Breda, Netherlands). Soil available phosphorus (AP) was extracted by the 0.5 M sodium bicarbonate solution and measured by spectrophotometry. Total phosphorus (TP) was measured by inductively coupled plasma spectrometry-MS after sample digestion using HClO_4_−H_2_SO_4_ solution. The reagents used above are analytical reagent (AR), and all the solutions were prepared using Millipore ultrapure water. Soil texture (i.e., particle composition, <0.002 mm as clay, 0.002~0.05 mm as silt and >0.05 mm as sand) was measured with a Mastersizer 3,000 laser particle size analyzer (Malvern Panalytical Ltd., United Kingdom).

### DNA Extraction and Illumina Sequencing of Fungal ITS Region

Soil genomic DNA was extracted from 0.5 g lyophilized soils in triplicate using PowerSoil DNA Isolation Kits (MO BIO laboratories, Carlsbad, United States) following the protocols described previously (Wang et al., 2016). The purity and concentration of the extracted DNA were examined using gel electrophoresis and NanoDrop ND-1000 spectrophotometer (NanoDrop Technologies, United States). Primer pairs ITS1F (5'-CTTGGTCATTTAGAGGAAGTAA-3') and ITS2 (5'-GCTGCGTTCTTCATCGATGC-3') with a 6 bp barcode were used to amplify the internal transcribed spacer (ITS) region as previously described (Martin and Rygiewicz, 2005). Amplicons were purified using the Wizard^®^ SV Gel and PCR Clean-Up System (Promega, San Luis Obispo, United States), then equimolarly mixed, and used for paired-end 250 bp sequencing on an Illumina MiSeq sequencer (Illumina Inc., San Diego, United States).

### Bioinformatics Analysis

For raw paired-end fastq reads, adaptors at the beginning of forward/reverse reads were trimmed off using USEARCH (Edgar, 2010). A maximum expected error (ee) was set as 0.5 for quality filtering of the merged reads. Dereplication was performed on those high-quality reads before operational taxonomic unit (OTU) clustering, and 100% identity OTU (denoised sequences, *i.e.,* zOTU) was gained by denoising (error correction) the amplicon reads using UNOISE3 (Edgar, 2016). Representative sequences were annotated against the UNITE database (Kõljalg et al., 2005) using a comprehensive taxonomic classification algorithm in CONSTAX (Gdanetz et al., 2017), and taxonomically ambiguous OTUs were further blasted against the MycoCosm database (Grigoriev et al., 2013). About 2.5 million of high-quality ITS reads were mapped (average 13,048 reads *per* sample), and a normalization procedure was performed and retained 8,604 sequences *per* sample before further analysis. After removing chimeras and singletons, all the reads were clustered into 3,778 OTUs at 100% sequence identity. Rarefaction curve indicated the sequencing effort was sufficient in representing the majority of fungal ITS groups (Supplementary Figure S2). Resultant OTU table was converted into the BIOM file and imported into QIIME (Caporaso et al., 2010) for diversity calculation. Alpha diversity (i.e., OTU richness) and the abundance-based beta diversity, Bray-Curtis matrices, were calculated using *alpha_diversity.py* and *beta_diversity.py* scripts in QIIME, respectively. Ordination of the beta diversity matrices was performed using the *nmds.py* script in QIIME. The representative sequences of OTUs were assigned to different functional groups (trophic mode and guild) using the open annotation tool FUNGuild (Nguyen et al., 2016).

### Determination of Generalist and Habitat Specialist Fungi

The concepts of microbial generalist and specialist were defined based on the niche breadth index (*B*-value; Levins, 1968; Pandit et al., 2009; Logares et al., 2013),

$$B_{j}=\frac{1}{\sum_{i=1}^{N}P_{ij}^{2}}$$

in which *B_j_* is the niche breath of OTU_j_ and *P_ij_* indicates the portion of OTU_j_ in sample i. B-values measured both abundance and occurrence. OTUs with a higher *B*-value would be considered as generalist taxa while OTUs with a lower *B*-value as habitat specialist. The niche breath is initially based on the resource gradients/environmental tolerance (Levins, 1968); in this definition, we used niche breath *via* presence in samples as an indirect approach to prevent potential circularity of inferences (Pandit et al., 2009).

We first calculated the *B*-values of OTUs across all the samples and filtered out those with mean relative abundance lower than 2 × 10^−5^ (Pandit et al., 2009). Those with *B*-values higher than 17 were regarded as generalist candidates (the value was set because it lays within the outlier area of the *B*-value distribution, detailed information shown in Supplementary Figure S3). Candidates with a frequency of occurrence more than 50% across all the samples were finally considered as generalists. For habitat specialist fungi determination, we first calculated the *B*-values of OTUs across the samples within individual regions and chose taxa with *B*-values lower than 12, 17, and 17 as specialist candidates in subtropical, warm temperate, and temperate regions, respectively (these values were set because they lay by the outlier area of the *B*-value distribution, as shown in Supplementary Figure S4). Only candidates with the occurrence higher than four were regarded as habitat specialists (this value was set because it was used in network analysis to eliminate spurious correlations).

### Fungi Coexisting Network Construction

The Cytoscape plugin CoNet was employed to calculate the significant correlations between fungal OTUs (Shannon et al., 2003; Faust et al., 2012). Prior to calculation, OTUs with less than four occurrences and 10 reads were excluded to minimize spurious correlations of rare taxa. Five different correlation algorithms, including Spearman correlation, Pearson correlation, Bray-Curtis distance, Kullback-Leibler distance, and mutual information were calculated, and correlations with only one algorithm supported were excluded (Hu et al., 2016). The Brown *p*-value combination algorithm was used for *p*-value merging. The Benjamini-Hochberg (BH) multiple test with 1,000 bootstraps and permutations was used to control the false positive rate (*p* < 0.001; Weiss et al., 2016). Topological properties of the resultant network, including node degree of connectivity, closeness, and betweenness centrality, were calculated in Gephi (Bastian et al., 2009). Relationships between B-value and closeness/betweenness centrality were measured using linear regression models and plotted using the R^1^ package *ggplot2* (Ginestet, 2011). To measure the importance of node in structuring the network, another topological property eigenvector centralities (EC) was calculated as previously described (Iyer et al., 2013; Wang et al., 2015).

### Structural Equation Modeling

Structural equation model (SEM) was used to evaluate the contributions of generalists and habitat specialists to the coexistence patterns across large spatial scales (Grace, 2006). Following variables were included in the analysis: climatic factors, including mean annual temperature (MAT) and mean annual precipitation; soil texture, including sand and clay content (silt was not included because it had strong collinearity with the other two); and carbon, including SOC and DOC, nitrogen, including TN, NH_4_^+^-N, and NO_3_^−^-N, and phosphorus, including TP and AP. Mantel test was performed to calculate the correlations among those factors aforementioned, and a covariance matrix of these factors was used as the input data. Adequate model fits were determined according to a non-significant chi-square test (*p* > 0.05), high goodness fit index (> 0.90), low Akaike information criterion value, and root-square-mean error of approximation (< 0.05) as previously described (Delgado-Baquerizo et al., 2016). SEM analyses were conducted using AMOS 17.0.2, and Mantel correlations were performed using the *vegan* package in R. Standardized total effects of spatial, climatic, and edaphic factors on the generalist and habitat specialist fungi were calculated in SEM (Grace, 2006).

## Results

### Community Composition of Soil Fungi in Cropping Systems Across Different Regions

**Figure 1:**
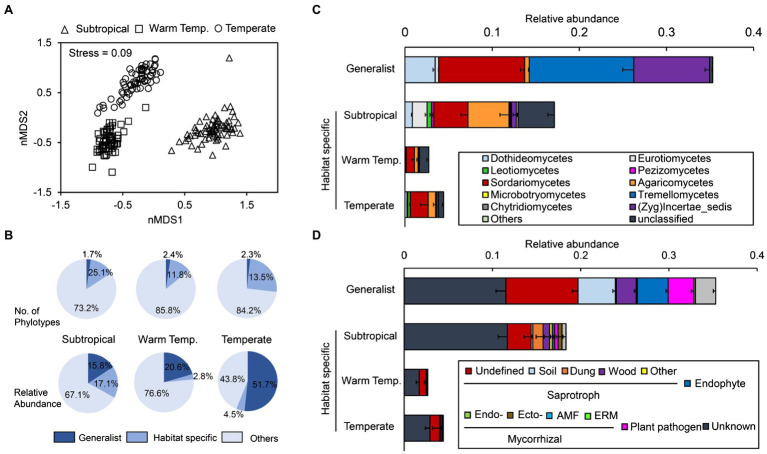
Fungal community compositions in arable soils across subtropical, warm temperate, and temperate areas. **(A)** Fungal community variance at the operational taxonomic unit (OTU) level. The non-metric multidimensional scaling was performed on the Bray-Curtis dissimilarity matrix. **(B)** Portion of the generalist and specialist fungi in different areas. **(C,D)** The taxonomic (the averaged relative abundance of fungal taxa at the order level) and functional (the averaged relative abundance of different functional guilds) composition of generalist and specialist fungi in different areas. Warm temp., warm temperate; Zyg, Zygomycota; AMF, arbuscular mycorrhizae fungi; and ERM, ericoid mycorrhiza.

Three phyla, including Ascomycota, Basidiomycota, and Zygomycota, dominated the cropland soil fungal community, and they accounted for 94.4% of the relative abundance (Supplementary Figure S5a). Rozellomycota was significantly enriched in the subtropical (0.5%) and warm temperate cropland soils (0.7%) but not in temperate soils (0.03%). Overall, fungal taxonomic composition was more variable between different regions than within the same region. Fungal communities in subtropical areas were different from those in the other two regions along the first axis, and communities in temperate regions were clearly different from others along the second axis (Figure 1A).

Results showed that on average, 63.6% of the fungi could be classified into specific trophic mode in FUNGuild, and three functional groups could be identified (Supplementary Figure S5b). Briefly, saprotrophic fungi dominated the community (36.7%, those break down dead host cells), followed by pathotrophic fungi (14.6%, those gain nutrients at the cost of host cell fitness and cause disease), and symbiotrophic fungi (12.3%, those exchange resources with host cells).

### Generalist and Specialist Fungi in Different Regions

Of the 3,778 OTUs retrieved, 44 OTUs (1.2%) were identified as generalist taxa (defined as those with more than 50% occurrence across all samples and high niche breath, i.e., >17). Approximately, 629 (16.7%), 214 (5.7%), and 254 (6.7%) fungal OTUs were identified as habitat specialists (those dominated only in individual bioclimatic region) in subtropical, warm temperate, and temperate cropland soils, respectively. However, on average, the generalists accounted for 35.3% of all ITS sequences across regions, and the specialists accounted for 17.1, 2.8 and 4.5% of the relative abundance of fungal communities within subtropical, warm temperate, and temperate regions, respectively. In other words, a few generalists dominated fungal communities cropland soils across bioclimatic regions (Figure 1B).

Most generalist taxa were Ascomycota (33 of the 44), and they were further assigned as Sordariomycetes, Dothideomycetes, and Eurotiomycetes (Figure 1C). Most highly frequent generalists (we defined here as > 85% frequency) could be found in the phylum Ascomycota, e.g., *Phoma* (89.9%), *Alternaria* (89.3–93.4%), and *Fusarium* (87.3–100%). Specifically, *Fusarium oxysporum* was the only generalist detected in every individual sample (100% frequency), and its average relative abundance was 3.1% across all the samples (Supplementary Table S1). Four generalist OTUs belonged to Basidiomycota, and they were further assigned as Tremellomycetes, and *Guehomyces pullulans* had the highest relative abundance across all samples (5.34%). All the six Zygomycota generalist OTUs belonged to the genus *Mortierella*.

Besides the unassigned and others, generalist fungi mainly included three functional groups, including saprotroph, endophyte, and plant pathogens (Figure 1D). Unlike generalists, only a few habitat specialists were assigned as saprotroph (mostly undefined) and most specialists are functionally unknown. Within each climate region, generalist fungi were more strongly related with the community functional composition (determined using the Bray-Curtis distance on functional guild composition) than habitat specialists (Supplementary Figure S6).

### Generalist vs. Specialist Taxa in Structuring Fungal Ecological Networks

**Figure 2:**
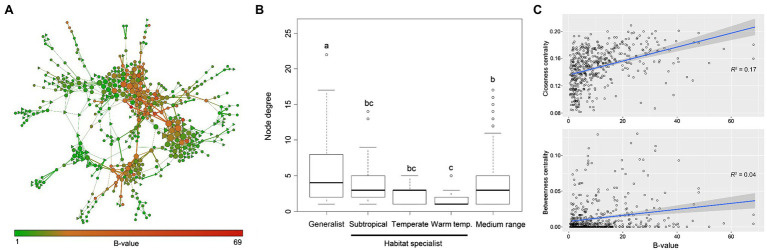
Structure and topology of the fungal ecological networks. **(A)** Coexisting network of fungal OTUs in arable soils across large spatial scales. Nodes indicate OTUs, and edges indicate significant (*p* < 0.001) and robust (*p* > 0.6) correlations between OTUs. The size of individual OTU is proportional to its importance in the network, i.e., eigenvector centrality. Width of the edge indicates the robustness of the relationship, and nodes are colored according to the B-values of OTUs. Hexagons indicate generalist fungi, and triangles indicate habitat specialist fungi. **(B)** Node degree distribution of fungal OTUs in the coexisting network. Habitat specialist fungi in subtropical, warm temperate, and temperate biomes were plotted separately. Medium range indicates OTUs that are neither generalist nor habitat specialist. Lines in the boxes indicate median, while top and bottom of the box indicate first and third quartiles, respectively. Whiskers mean 1.5 interquartile range. One-way ANOVA was performed to test the significance among groups (*F* = 4.97, *p* < 0.05), and different lower-case letters indicate statistical difference. **(C)** Relationships between B-value and closeness/betweenness centrality of nodes in the network. Closeness centrality measures the centrality within modules, and higher values mean that the node has more linkages with others around; betweenness centrality measures the centrality among modules, and higher values mean that the node could be a connector among modules. Shadow indicates 95% confidence range.

The fungal ecological network consisted of 485 nodes (fungal OTUs) and 952 edges (strong correlations between OTUs). Overall, 84% of the generalist fungi were involved in the fungal coexistence network, while only 17.0%/6.1%/5.5% habitat specialists were included. Even though the medium-range fungi (neither generalist nor habitat specialist) occupied most of the nodes in the network, generalist fungi were found to have higher within-module centrality (Supplementary Figure S7). They usually had greater importance (i.e., higher EC value) than habitat specialist fungi within the network (Figure 2A). Generalist fungi had significantly higher degree of connectivity (*p* < 0.05) than habitat specialist fungi or medium-range fungi in the network, and specialist fungi in warm temperate areas had the lowest degree (Figure 2B). The top four OTUs with the highest closeness centrality were identified as generalist (OTU58, OTU7, OTU26, and OTU45). Linear regression models further revealed that node B-values increased with the node closeness centrality (*r*^2^ = 0.17) but not with betweenness centrality (*r*^2^ = 0.04), indicating that generalist fungi would possibly be a module hub rather than a connector among modules in the network (Figure 2C).

### Factors Driving the Fungal Coexistence Pattern

**Figure 3:**
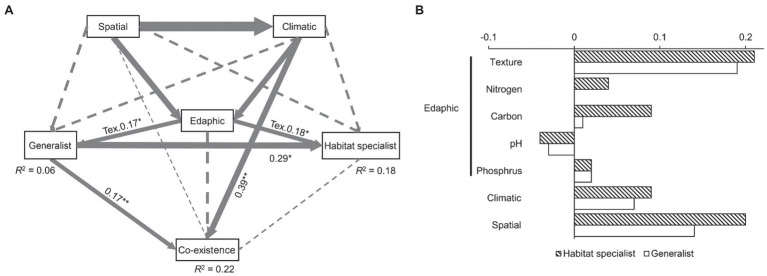
Factors driving the fungal generalists/habitat specialists and the coexisting pattern **(A)**, and the standardized total effects of spatial, climatic, and edaphic factors on generalist/habitat specialist fungi **(B)**. Edaphic factor includes soil pH, texture, total nitrogen, soil organic carbon, and available phosphorus. Solid arrows indicate significant relationships, while dash lines, non-significant. The width of the arrows represents the strength of the influence. Goodness-of-fit statistics are evaluated as follows: chi-square = 46.5, *p* = 0.89, DOF = 14, RMSEA = 0.02, AIC = 84.55, and GFI = 0.98. Significant level: ^*^p < 0.05, ^**^p < 0.01. DOF, degrees of freedom; RMSEA, root-mean-square error of approximation; AIC, Akaike information criterion; GFI, goodness fit index; and Tex, texture.

Our structural equation models could explain 22% of the fungal coexistence pattern (Figure 3A). It is interesting that even though this model explained much less generalist fungal variation (merely 6%) than the specialist variation (18%), generalist fungi had a significant and direct effect (0.17, *p* < 0.01) on fungal coexistence pattern while habitat specialist had no significant effect. Soil texture was the only edaphic factor that significantly affected both generalist and habitat specialist fungi. Climatic condition had a significant effect on the fungal coexistence pattern. The standardized total effects from the SEM revealed that habitat specialist fungi were more sensitive to space isolation, climate, and edaphic factors (including soil texture, carbon, nitrogen, soil pH, and phosphorus) than the generalists. Specifically, generalists were much less sensitive to soil organic and nitrogen content than habitat specialist fungi (Figure 3B).

## Discussion

### Generalist Taxa Dominate the Fungal Communities Across Agricultural Ecosystems

Generalists occupied a small portion of the OTUs in cropland soil fungal communities (1.2%), and all of them were classified as dominant taxa. Like the previous finding for bacteria (Delgado-Baquerizo et al., 2018), these small numbers of fungal taxa dominated the fungal populations (35.2% of the relative abundance). On the other hand, the proportion in this study is much higher than the result of less than 0.1% fungal taxa identified as dominants in fungal community from a global study on fungi in natural habitats (Egidi et al., 2019). This discrepancy could be partly attributed to the difference in habitat heterogeneity between natural and cropland habitats.

Previous studies showed that generalist taxa likely play important roles in sustaining the ecosystem function; for example, bacteria generalists are more important than specialists in regulating the carbon cycling in marine ecosystem (Mou et al., 2008). Our study suggests that generalists are probably the main contributors to nutrient availability and plant pathogenicity in agricultural ecosystems. Generalist fungi are closely related to the functional composition of the community (Supplementary Figure S6), and nearly half of the generalists are saprotrophic. Saprotrophic fungi are usually regarded as the most important decomposers in natural ecosystems, like forest, while our results indicated that in cropland ecosystems, they might also be important contributors to soil carbon source by decomposing crop litters. About 10% of the generalist fungi are classified as plant pathogens, while by contrast ~0.3% fungal pathogens was detected at the whole community level (Supplementary Figure S5b). *F. oxysporum* is likely to be a plant endophyte, a soil saprophyte, or an important plant pathogen in agricultural ecosystems (Cao et al., 2016). In our study, one of the most important generalists *F. oxysporum* dominated in all the samples examined, which indicated *F. oxysporum* as an important key fungus in cropland soils. Also, potential plant pathogens *Phoma* and *Alternaria* (Maude et al., 1984) were widely distributed and dominated the fungal community in cropland soil. These results indicate that generalists might be important in the pathogenicity process in cropland ecosystems. Besides, generalists also include potential symbiotic fungi that can benefit the crops. In this case, several Mortierella OTUs were identified as generalists (Supplementary Table S1). Mortierella species were reported as important plant growth-promoting fungi (PGPF) that produced plant hormones (Li et al., 2018); they were also reported to be adapted to a wide range of environmental gradient in cropland soils (Grządziel and Gałązka, 2019). Therefore, these beneficial generalists might be good candidates for PGPF screening. We agreed that the sequencing platform used here (Illumina MiSeq) did not provide high resolution to identify OTUs at the strain level, but these results provided observational evidence that generalist taxa play important roles in sustaining soil functions in cropping systems.

### Biogeographic Distribution and the Ecological Network of Soil Fungi

Generalist taxa have larger contribution to overall biogeographic patterns than habitat specialists in marine environment (Lindh et al., 2016). The two groups construct different relationships with other microbes, which could be separated by their ecological preference. The coexisting network provides an intuitive perspective on microbial relationships and ecological preference of different microbial taxa. Through the large-scale (over 2,000 kms) study on cropland soil fungal communities, our findings support the hypothesis that the same generalist taxa are important components of ecological networks. This result is inconsistent with Barberán’s work on soil bacterial network (Barberán et al., 2012), in which the generalists were found to be less connected with each other than specialists. Such difference might be due to the habitat heterogeneity, since their samples covered variety of ecosystems while ours only focused on managed ecosystems. In our study, we found that generalist fungi are mostly module hubs, and they are more important than habitat specialist taxa or other groups in shaping the structure of fungal ecological networks across large spatial scales. In other words, generalist taxa play critical roles in supporting the structure of fungal ecological networks in cropland soils.

### Generalist and Habitat Specialist Fungi Respond Differently to Edaphic and Climatic Variables

Generalists remain unchanged across environmental conditions, and hence, they are difficult to be predicted by other environmental factors. Our results revealed that only small proportion of the variation in generalist fungi could be predicted. This finding is probably related to the fact that these taxa are present in most soil samples, and hence, environmental variability is weak in predicting the distribution of generalist taxa. Therefore, our results suggest that generalist taxa (Supplementary Table S1) can be considered as the core soil fungal microbiome for cropland soils. These taxa potentially include highly resistant taxa that can thrive under different environmental conditions. For example, according to a study in Amazon rainforest, generalist fungi are strongly resistant to land use change (Mueller et al., 2016). In face of changing environmental moisture, generalists would be more tolerant and could adapt to a broader range of water potential than specialists (Lennon et al., 2012). In studies on managed systems, incorporation of management data (which we were not able to collect here), including nutrients input, fungicide, and cropping history, would benefit our interpretation on fungal community characteristics and their response.

Our SEM analysis provided important evidence that across large spatial scales, generalist fungi significantly affected the fungal ecological network and remained unchanged in response to climatic and edaphic factors than habitat specialists. Soil nitrogen and carbon are the two most typical edaphic factors that mostly driven by agricultural practices (especially fertilization), and they are known to have strong effect on fungal communities (Sun et al., 2016). In our case, soil nitrogen and carbon were found to have contrasting effect on generalists and specialists (Figure 3B), indicating that generalist fungi are less sensitive to soil factor changes that determined by human activities. Generalist fungi are tolerant to these changes, while habitat specialist taxa tend to be sensitive to disturbance. Therefore, prevalence of fungal taxa in agricultural ecosystems might be largely dependent on the tolerance of generalist fungi to human activities.

## Conclusion

Taken together, our results provided evidence that a few generalist taxa (~1%) dominated soil fungal communities in croplands across large spatial scales, and these taxa were key components to shape the fungal community structure. These findings suggest that knowledge of generalists can provide important implication for understanding the ecological preference of fungal groups in cropland systems.

## Data Availability Statement

The datasets presented in this study can be found in online repositories. The names of the repository/repositories and accession number(s) can be found at: https://www.ebi.ac.uk/ena, ERP115241.

## Author Contributions

J-ZH, J-PS, and L-MZ designed the research. J-ZH, J-PS, W-XW, Y-TF, L-LH, and J-TW lead the field work. J-PS and L-MZ lead the laboratory work. J-TW and MD-B performed the data analysis. J-TW, MD-B, BKS, J-ZH, J-PS, and H-WH interpreted the results and wrote the manuscript in close consultation from all authors. All authors contributed to the article and approved the submitted version.

### Conflict of Interest

The authors declare that the research was conducted in the absence of any commercial or financial relationships that could be construed as a potential conflict of interest.

## Footnotes

^1^https://www.r-project.org/
