# Supplementary material for: Generalist Taxa Shape Fungal Community Structure in Cropping Ecosystems
Source: Front Microbiol. 2021 Jul 9;12:678290. doi: 10.3389/fmicb.2021.678290 (PMC8299105; doi:10.3389/fmicb.2021.678290)
Supplement: Supplementary file 1 [file Data_Sheet_1.docx]

**Running Title: Generalists shape fungal community structure**

**Title: Generalist taxa shape fungal community structure in cropping ecosystems**

Jun-Tao Wang^1,2#^, Ju-Pei Shen^1,3#^, Li-Mei Zhang^1,3^, Brajesh K. Singh^2,4^, Manuel Delgado-Baquerizo^5^, Hang-Wei Hu^6,9^, Li-Li Han^1^, Wen-Xue Wei^7^, Yun-Ting Fang^8^, Ji-Zheng He^1,6,9*^

1 State Key Laboratory of Urban and Regional Ecology, Research Center for Eco-Environmental Sciences, Chinese Academy of Sciences, Beijing 100085, China.

2 Hawkesbury Institute for the Environment, Western Sydney University, Penrith, New South Wales 2751, Australia.

3 University of Chinese Academy of Sciences, Beijing 100049, China

4 Global Centre for Land-Based Innovation, Western Sydney University, Penrith South DC, NSW 2751, Australia.

5 Cooperative Institute for Research in Environmental Sciences, University of Colorado, Boulder, CO 80309, USA.

6 Faculty of Veterinary and Agricultural Sciences, the University of Melbourne, Parkville, Victoria, Australia.

7 Key Laboratory of Agro-ecological Processes in Subtropical Region, Institute of Subtropical Agriculture, Chinese Academy of Sciences, Changsha, China

8 CAS Key Laboratory of Forest Ecology and Management, Institute of Applied Ecology, Chinese Academy of Sciences, Shenyang 110164, China

9 Key Laboratory for Humid Subtropical Eco-geographical Processes of the Ministry of Education, Fujian Normal University, Fuzhou 350007, China

#These authors contributed equally to this article.

For correspondence:

*Ji-Zheng He, Tel. (+86)-10-62849788, Fax: (+86)-10-62923563, E-mail: [jzhe@rcees.ac.cn](mailto:jzhe@rcees.ac.cn)


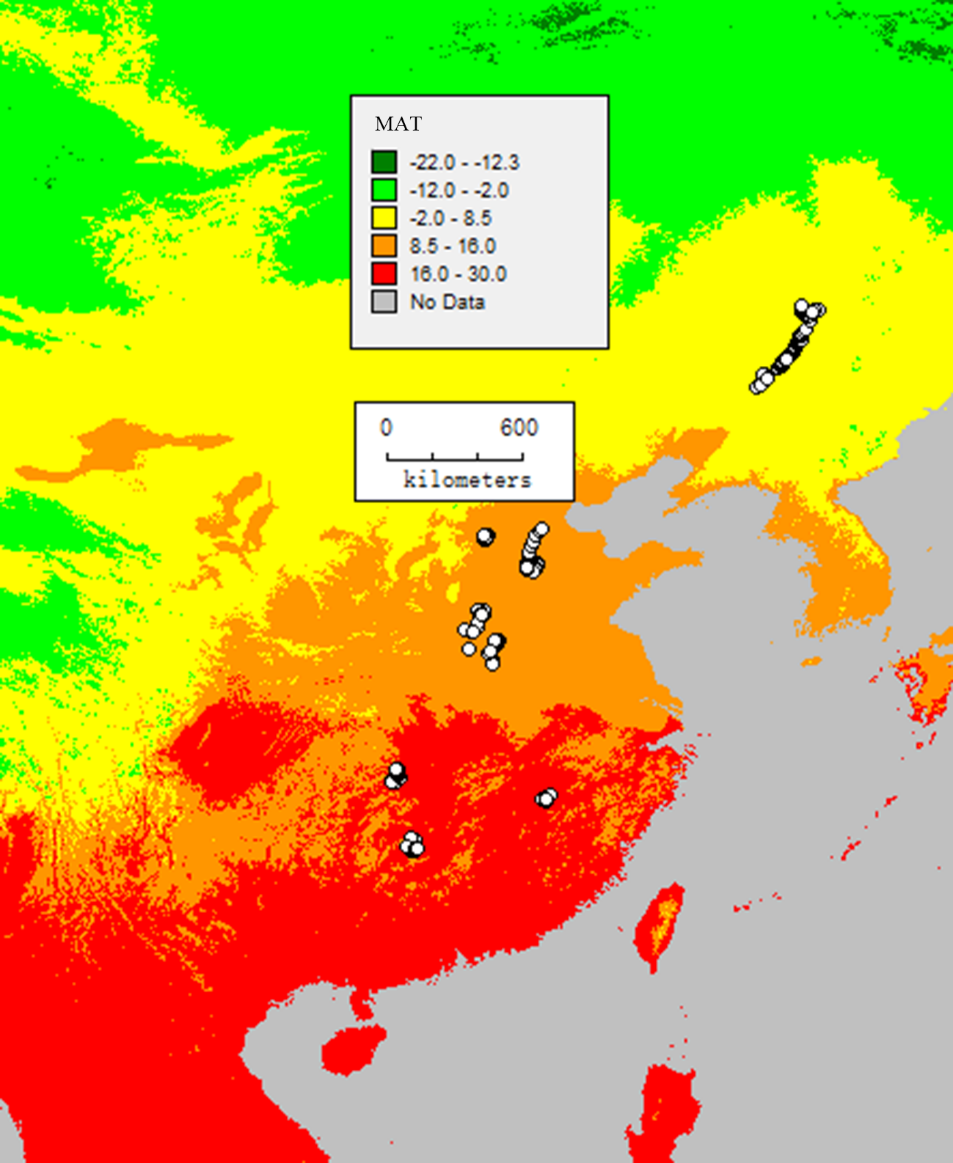


**FIGURE S1 Map of the sampling locations across subtropical, warm temperate and temperate areas in eastern China**. MAT indicates Mean Annual Temperature, and the map was generated using DIVA-GIS (http://www.diva-gis.org).


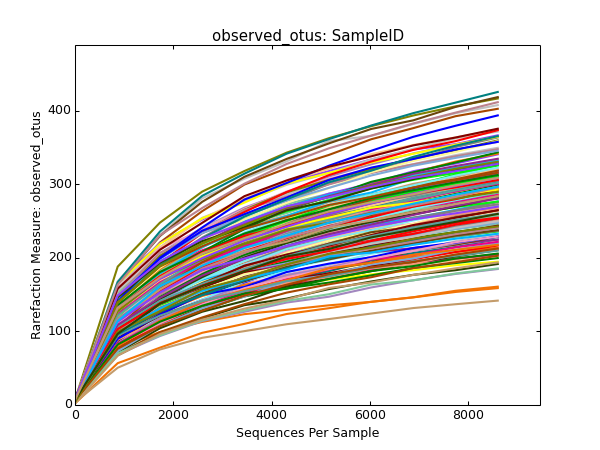


**FIGURE S2 Rarefaction curves of the fungal OTU richness** (*i.e.* observed species) at the resampling depth of 8,604 reads *per* sample.


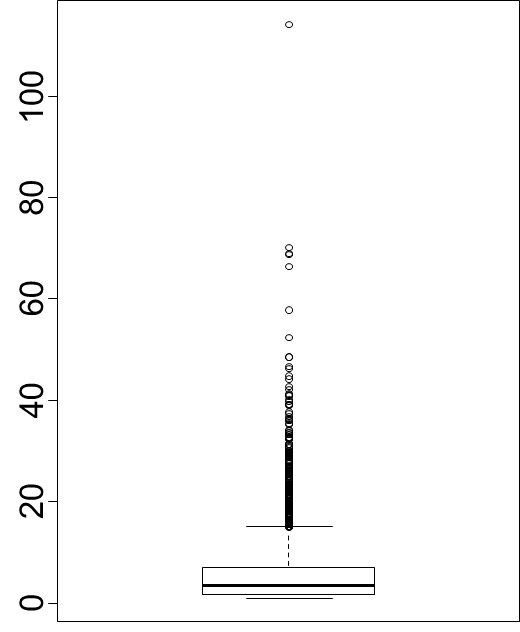


**FIGURE S3. B-values distribution of fungal OTUs across all the samples.**


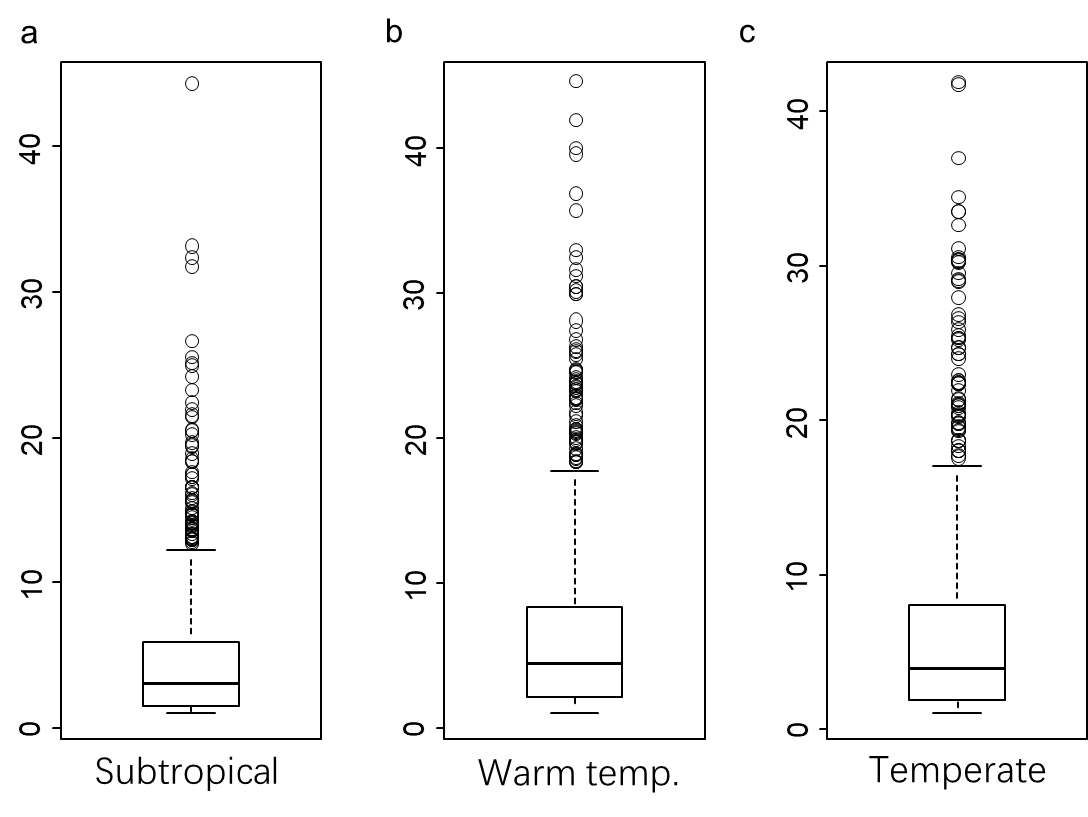


**FIGURE S4. B-values distribution of fungal OTUs in subtropical, warm temperate and temperate arable soils.** Warm temp. is short for Warm temperate.


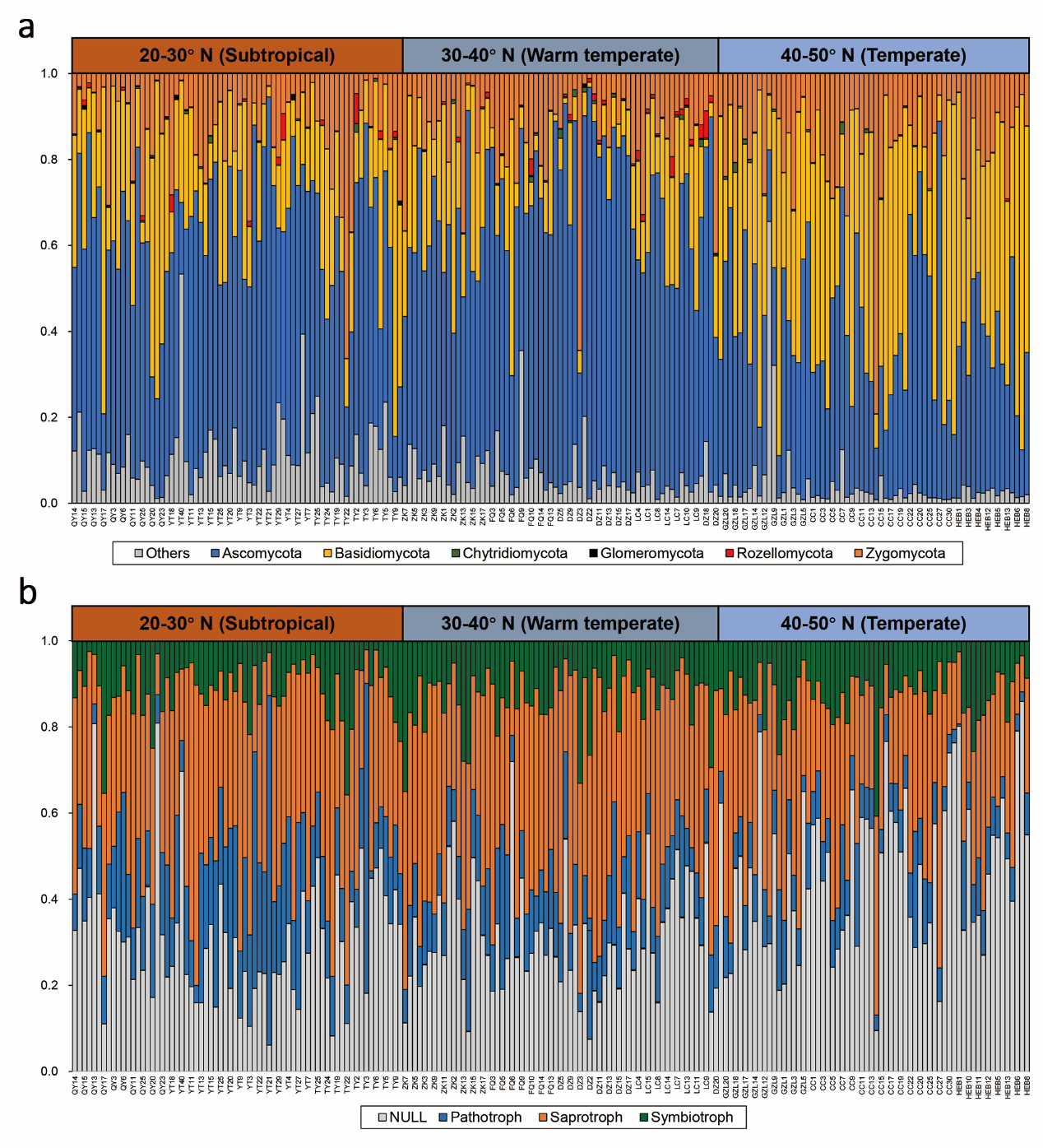


**FIGURE S5 Community composition of fungi in arable soils across subtropical, warm temperate and temperate areas**. Taxonomic information was provided by coloring different phyla (a), while function-related information was provided *as per* the trophic mode (b) by the FUNGuild annotation. Pathotroph indicates fungal taxa which receive nutrients at the expense of the host cells and causing disease. Saprotroph indicates fungal taxa which receive nutrients by breaking down dead host cells. Symbiotroph indicates fungal taxa which receive nutrients by exchanging resources with host cells. Relative abundance of each trophic guild was calculated using the portion of sequence counts of individual guild. Taxa with more than one guild were evenly divided to individual guild in calculation (e.g., an OTU with a relative abundance of 10% and identified as Pathotroph-Symbiotroph will be further divided as 5% Pathotroph and 5% Symbiotroph).





**FIGURE S6 Relationships between fungal trophic mode composition and common (a,b,c)/habitat specific fungi (d,e,f) in different areas.** a,d - subtropical biome; b,e - warm temperate biome; c,f - temperate biome. Mantel correlation was performed using the Pearson co-efficient with 999 permutation.


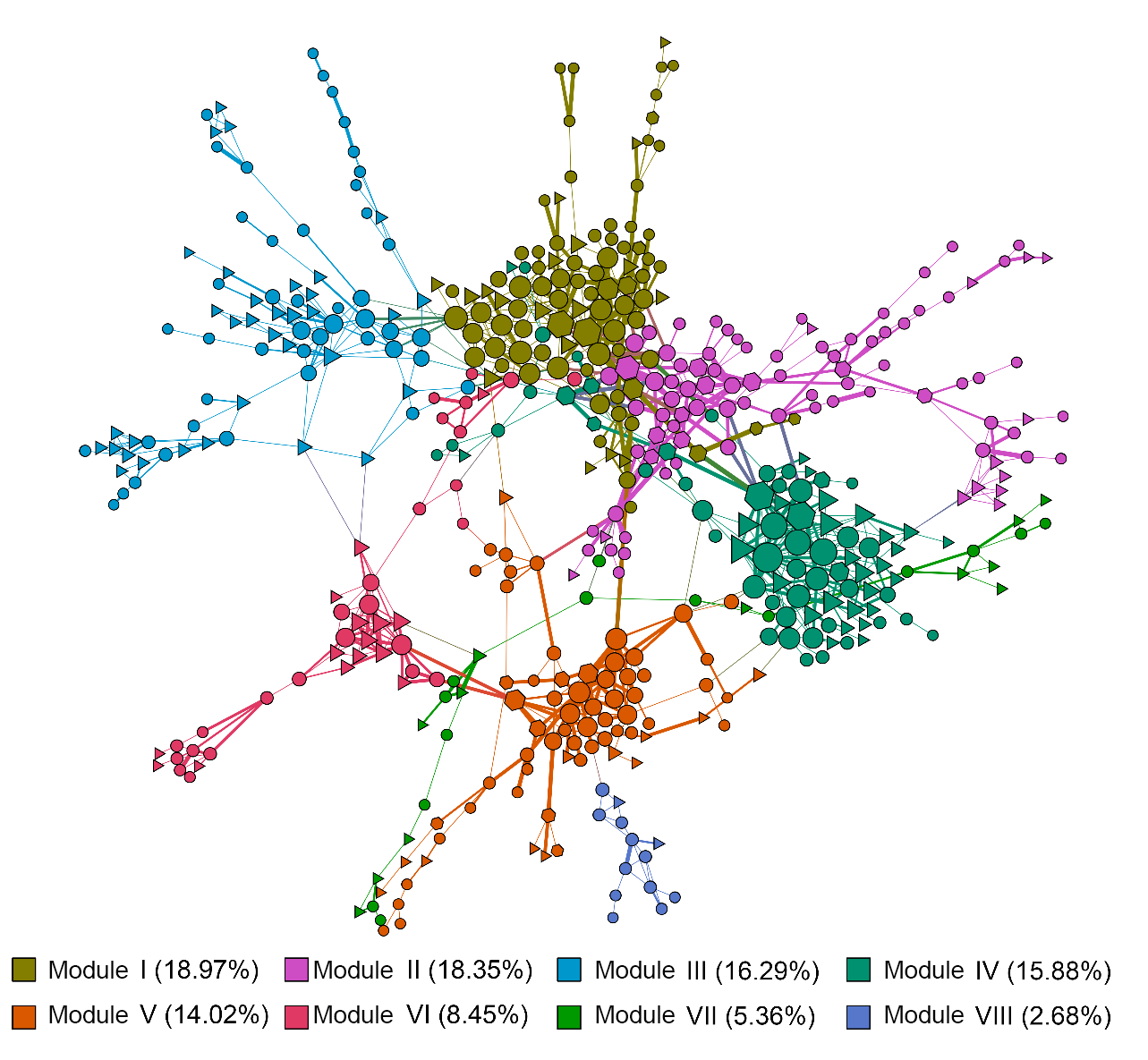


**FIGURE S7 Co-existing network of fungal OTUs in arable soils across large spatial scales.** Nodes indicate OTUs and edges indicate significant (*p* < 0.001) correlations between OTUs. The size of individual OTUs is proportional to its importance in the network, i.e. eigenvector centrality; Width of the edge indicates the robustness of the relationship. The nodes are colored according to modularity class, as demonstrated in the legend. Hexagon indicates common fungi and triangle indicates habitat specific ones. The Modularity of the network is 0.78, indicating that the network is well structured.

Table S1 Taxonomy and functions of fungal generalists

| OTU ID | Phylum | Class | Taxonomy | Frequency | Mean Relative Abund. | TrophicMode | Guild |
| --- | --- | --- | --- | --- | --- | --- | --- |
| OTU79 | Asc | unassigned | NA | 76.65% | 0.52% | NULL | NULL |
| OTU160 |  |  | NA | 66.50% | 0.10% | NULL | NULL |
| OTU163 |  |  | NA | 64.47% | 0.19% | NULL | NULL |
| OTU15 |  | Dothideomycetes | *Phoma spp.* | 89.85% | 1.02% | Pathotroph Saprotroph | Plant Pathogen-Wood Saprotroph |
| OTU32 |  |  | *Alternaria spp.* | 93.40% | 0.45% | Pathotroph Saprotroph Symbiotroph | Animal Pathogen-Endophyte-Plant Pathogen-Wood Saprotroph |
| OTU29 |  |  | *Alternaria spp.* | 89.34% | 0.44% | Pathotroph Saprotroph Symbiotroph | Animal Pathogen-Endophyte-Plant Pathogen-Wood Saprotroph |
| OTU167 |  |  | *Parastagonospora nodorum* | 74.62% | 0.12% | NULL | NULL |
| OTU7 |  |  | *Parastagonospora* | 60.91% | 1.55% | NULL | NULL |
| OTU278 |  | Eurotiomycetes | Herpotrichiellaceae | 61.42% | 0.10% | Pathotroph Saprotroph | Animal Pathogen-Fungal Parasite-Undefined Saprotroph |
| OTU223 |  |  | *Exophiala equina* | 53.30% | 0.07% | Pathotroph Saprotroph | Animal Pathogen-Undefined Saprotroph |
| OTU185 |  |  | *Talaromyces spp.* | 71.57% | 0.23% | Saprotroph | Undefined Saprotroph |
| OTU159 |  | Sordariomycetes | NA | 57.87% | 0.13% | NULL | NULL |
| OTU26 |  |  | Hypocreales | 80.71% | 0.51% | Saprotroph | Undefined Saprotroph |
| OTU198 |  |  | Hypocreales | 59.39% | 0.09% | Saprotroph | Undefined Saprotroph |
| OTU367 |  |  | *Trichoderma spp.* | 54.82% | 0.05% | Saprotroph | Undefined Saprotroph |
| OTU232 |  |  | *Acremonium curvulum* | 55.84% | 0.06% | Pathogen Saprotroph Symbiotroph | Animal Pathogen-Endophyte-Fungal Parasite-Plant Pathogen-Wood Saprotroph |
| OTU141 |  |  | *Ilyonectria macrodidyma* | 53.30% | 0.10% | Saprotroph | Undefined Saprotroph |
| OTU9 |  |  | Nectriaceae | 96.95% | 1.68% | Saprotroph | Undefined Saprotroph |
| OTU72 |  |  | Nectriaceae | 75.13% | 0.29% | Saprotroph | Undefined Saprotroph |
| OTU154 |  |  | Nectriaceae | 69.04% | 0.10% | Saprotroph | Undefined Saprotroph |
| OTU336 |  |  | Nectriaceae | 64.47% | 0.04% | Saprotroph | Undefined Saprotroph |
| OTU31 |  |  | *Fusarium spp.* | 96.95% | 0.45% | Pathotroph Saprotroph | Plant Pathogen-Soil Saprotroph-Wood Saprotroph |
| OTU98 |  |  | *Fusarium spp.* | 56.85% | 0.17% | Pathotroph Saprotroph | Plant Pathogen-Soil Saprotroph-Wood Saprotroph |
| OTU63 |  |  | *Fusarium spp.* | 87.31% | 0.33% | Pathotroph Saprotroph | Plant Pathogen-Soil Saprotroph-Wood Saprotroph |
| OTU4 |  |  | *Fusarium oxysporum* | 100.00% | 3.05% | Pathotroph Saprotroph | Plant Pathogen-Soil Saprotroph-Wood Saprotroph |
| OTU54 |  |  | *Gibberella spp.* | 83.76% | 0.32% | Pathotroph | Plant Pathogen |
| OTU128 |  |  | *Gibberella zeae* | 77.66% | 0.12% | Pathotroph | Plant Pathogen |
| OTU57 |  |  | *Purpureocillium lilacinum* | 72.59% | 0.38% | Pathotroph | Fungal Parasite |
| OTU157 |  |  | Cephalothecaceae | 53.30% | 0.10% | Saprotroph | Fungal Parasite-Wood Saprotroph |
| OTU37 |  |  | *Chaetomium globosum* | 89.34% | 0.58% | Pathotroph Saprotroph Symbiotroph | Animal Pathogen-Dung Saprotroph-Endophyte-Epiphyte-Plant Saprotroph-Wood Saprotroph |
| OTU173 |  |  | Lasiosphaeriaceae | 49.75% | 0.15% | Saprotroph | Undefined Saprotroph |
| OTU58 |  |  | *Microdochium bolleyi* | 64.47% | 0.37% | Pathotroph Symbiotroph | Endophyte-Plant Pathogen |
| OTU169 |  |  | *Microdochium spp.* | 68.02% | 0.09% | Pathotroph Symbiotroph | Endophyte-Plant Pathogen |
| OTU97 | Bas | Agaricomycetes | *Ceratobasidium sp* | 64.97% | 0.53% | Pathotroph Saprotroph Symbiotroph | Endomycorrhizal-Plant Pathogen-Undefined Saprotroph |
| OTU3 |  | Tremellomycetes | *Guehomyces pullulans* | 86.80% | 4.13% | NULL | NULL |
| OTU1 |  |  | *Guehomyces pullulans* | 61.42% | 5.34% | NULL | NULL |
| OTU5 |  |  | *Cryptococcus aerius* | 69.54% | 2.52% | Pathotroph Saprotroph Symbiotroph | Fungal Parasite-Undefined Saprotroph |
| OTU2 | Zyg | Incertae_sedis | *Mortierella spp.* | 89.85% | 5.11% | Saprotroph Symbiotroph | Endophyte-Soil Saprotroph-Undefined Saprotroph |
| OTU162 |  |  | *Mortierella alpina* | 63.45% | 0.16% | Saprotroph Symbiotroph | Endophyte-Soil Saprotroph-Undefined Saprotroph |
| OTU45 |  |  | *Mortierella alpina* | 51.78% | 0.39% | Saprotroph Symbiotroph | Endophyte-Soil Saprotroph-Undefined Saprotroph |
| OTU6 |  |  | *Mortierella elongata* | 76.14% | 2.14% | Saprotroph Symbiotroph | Endophyte-Soil Saprotroph-Undefined Saprotroph |
| OTU19 |  |  | *Mortierella elongata* | 62.44% | 0.64% | Saprotroph Symbiotroph | Endophyte-Soil Saprotroph-Undefined Saprotroph |
| OTU80 |  |  | *Mortierella spp.* | 59.39% | 0.26% | Saprotroph Symbiotroph | Endophyte-Soil Saprotroph-Undefined Saprotroph |
| OTU187 | unknown |  | NA | 85.79% | 0.15% | NULL | NULL |

Abbreviations: Abund., Abundance; Asc, Ascomycota; Bas, Basidiomycota; Zyg, Zygomycota.
